# Transcription factor Six2 mediates the protection of GDNF on 6-OHDA lesioned dopaminergic neurons by regulating Smurf1 expression

**DOI:** 10.1038/cddis.2016.120

**Published:** 2016-05-05

**Authors:** J Gao, X-y Kang, S Sun, L Li, B-l Zhang, Y-q Li, D-s Gao

**Affiliations:** 1 Department of Anatomy and Histology, The Fourth Military Medical University, Xian 710003, Shanxi, China; 2 Department of Neurobiology and Anatomy, Xuzhou Key Laboratory of Neurobiology, Jiangsu Key Laboratory of New Drug Research and Clinical Pharmacy, Xuzhou Medical College, Xuzhou 221004, Jiangsu, China

## Abstract

Glial cell line-derived neurotrophic factor (GDNF) has strong neuroprotective and neurorestorative effects on dopaminergic (DA) neurons in the substantia nigra (SN); however, the underlying molecular mechanisms remain to be fully elucidated. In this study, we found that the expression level of transcription factor Six2 was increased in damaged DA neurons after GDNF rescue *in vivo* and *in vitro*. Knockdown of Six2 resulted in decreased cell viability and increased the apoptosis of damaged DA neurons after GDNF treatment *in vitro*. In contrast, Six2 overexpression increased cell viability and decreased cell apoptosis. Furthermore, genome-wide chromatin immunoprecipitation sequencing (ChIP-seq) indicated that Six2 directly bound to the promoter CAGCTG sequence of smad ubiquitylation regulatory factor 1 (Smurf1). ChIP-quantitative polymerase chain reaction (qPCR) analysis showed that Smurf1 expression was significantly upregulated after GDNF rescue. Moreover, knockdown of Six2 decreased Smurf1 expression, whereas overexpression of Six2 increased Smurf1 expression in damaged DA neurons after GDNF rescue. Meanwhile, knockdown and overexpression of Smurf1 increased and decreased p53 expression, respectively. Taken together, our results from *in vitro* and *in vivo* analysis indicate that Six2 mediates the protective effects of GDNF on damaged DA neurons by regulating Smurf1 expression, which could be useful in identifying potential drug targets for injured DA neurons.

Neurotrophic factors have recently emerged as promising therapeutic drugs for neurodegenerative diseases. Among these, glial cell line-derived neurotrophic factor (GDNF) has attracted special attention for its protective effects on injured dopaminergic (DA) neurons in Parkinson's disease (PD).^1, 2, 3^ Although enhancing GDNF signalling protects damaged DA neurons, little is known about its protective mechanisms.

Apoptosis has been implicated as one of the important mechanisms leading to DA neuronal death in the substantia nigra (SN) of PD patients.^4, 5^ Previous reports indicated that exogenous GDNF could improve the survival and reduce apoptosis of embryonic DA neurons.^6, 7^ Similarly, the combined application of GDNF and caspase inhibitor enhances the survival of grafted DA neurons.^8^ In lactacystin-induced DA neurodegeneration, GDNF significantly inhibits DA neuron apoptosis and reduces caspase-3 activation.^9^ We previously demonstrated that GDNF protects 6-hydroxydopamine (6-OHDA)-injured MN9D cells by upregulating Bcl-2 and Bcl-w expression.^10^ Those studies suggested that an anti-apoptotic effect was likely the primary protective mechanism of GDNF on damaged DA neurons. However, the precise mechanisms remain to be fully elucidated.

The transcription factor Six2 is a member of the sine oculis homeobox (SIX) family. It was previously identified as a critical regulator in normal kidney development.^11, 12^ Several recent lines of evidence have indicated that Six2 is also involved in organ development and some diseases.^13, 14, 15^ Meanwhile, studies have also indicated that Six2 has the function of anti-apoptosis, for example, downregulated Six2 induces metanephric mesenchyme cell apoptosis,^16^ and miR181 could also promote apoptosis by targeting Six2 *in vitro*.^17^ We previously observed aberrant Six2 expression in damaged DA neurons after GDNF rescue, which led us to hypothesise that GDNF upregulates Six2 to prevent damaged DA neurons from undergoing apoptosis.

In the present study, we provide evidence that Six2 is pivotal for GDNF protection in injured DA neurons. Knockdown of Six2 by lentiviral shRNA and Six2 overexpression decreased and increased the protection afforded by GDNF, respectively. Further screening of Six2 targets by genome-wide chromatin immunoprecipitation sequencing (ChIP-seq) identified the smad ubiquitylation regulatory factor 1 (Smurf1) as a specific target of Six2, and this inhibited the apoptosis of damaged DA neurons. This study may be of significant value to identify potential drug targets for injured DA neurons.

## Results

### GDNF rescues 6-OHDA-damaged DA neurons

**Figure 1:**
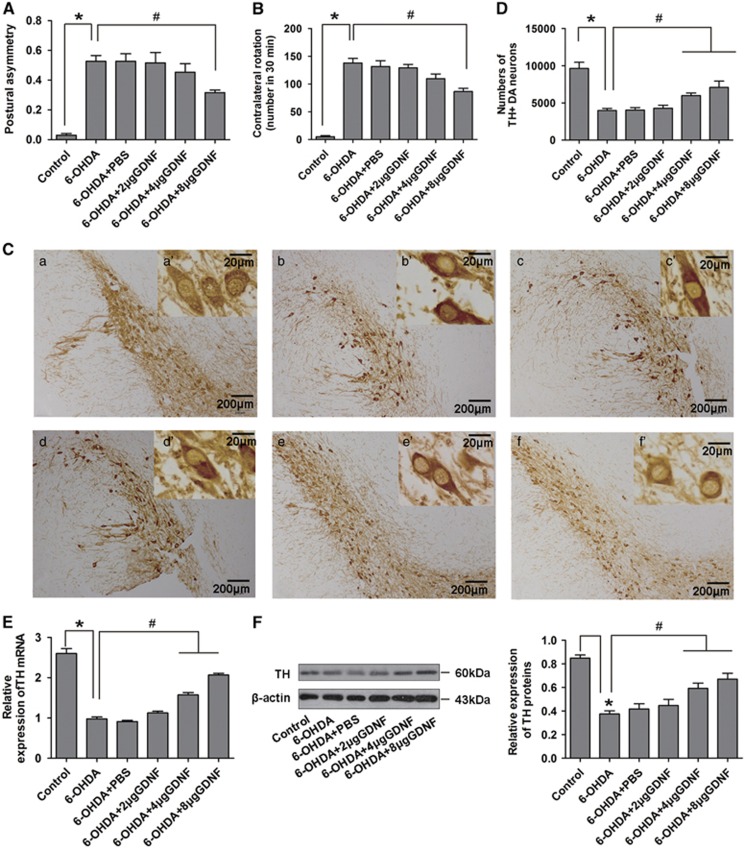
Effects of GDNF on DA neurons in SN of PD rats. (**A** and **B**) Results of postural asymmetry and apomorphine-induced rotation of rats in all groups (16 *μ*g 6-OHDA for 2 w followed by different doses of GDNF for 1 w). Bar graphs are shown as means±S.E.M. (*n*=10). (**C**) Immunohistochemistry analysis of TH^+^ DA neuron after GDNF rescue in SN of PD rats (16 *μ*g 6-OHDA for 2 w followed by different doses of GDNF for 1 w). (a) Control group, (b) 6-OHDA group, (c) 6-OHDA+PBS group, (d) 6-OHDA+2 *μ*g GDNF group, (e) 6-OHDA+4 *μ*g GDNF group, (f) 6-OHDA+8 *μ*g GDNF group; scale bars (in a–f) equals 200 *μ*m. a'–f' are higher magnification images in a–f, scale bars equals 20 *μ*m. (**D**) Counting of the above TH^+^ DA neurons in the SN of PD rats after GDNF rescue. (**E** and F). TH mRNA and protein expression levels in damaged DA neurons after GDNF rescue in the SN of PD rats (16 *μ*g 6-OHDA for 2 w followed by different doses of GDNF for 1 d). Bar graphs are shown as means±S.E.M. (*n*=5). *P<0.05 *versus* control group, ^#^*P*<0.05 *versus* the 6-OHDA group. The statistical analysis was carried out using one-way ANOVA followed by *post hoc* Newman–Keuls tests

To verify that GDNF could protect damaged DA neurons, we generate a rat PD model using 6-OHDA. The posture asymmetry experiment indicated that the frequency of head turning to the lesioned side improved after GDNF rescue (Figure 1A), in addition, apomorphine-induced rotation test confirmed that the motor abilities of PD rats were significantly improved, particularly in the 8 *μ*g GDNF groups (Figure 1B). To further verify the protection of GDNF on damaged DA neurons, the numbers of TH^+^ DA neurons in the SN were counted. The numbers of TH^+^ neurons were significantly increased in the 8 *μ*g GDNF group compared with the 6-OHDA group; however, cell numbers were only slightly increased in the 4 *μ*g GDNF group and almost unchanged in the 2 *μ*g GDNF group (Figures 1Ca–f and D), indicating a dose-dependent effect. We also examined TH expression in the SN, and found that TH dose-dependently increased after GDNF rescue (Figures 1E and F). These results suggest that GDNF significantly protects damaged DA neurons in the SN of PD rats.

**Figure 2:**
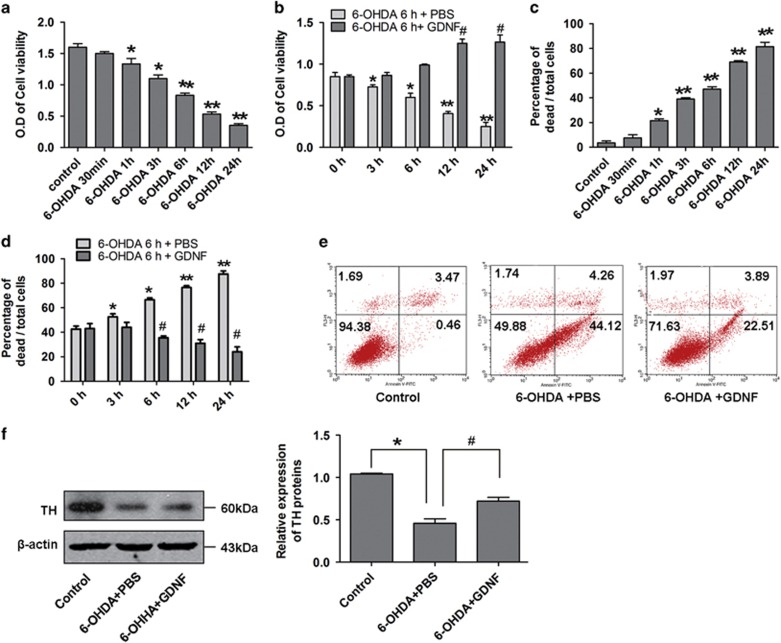
Effects of GDNF on MES23.5 DA neurons *in vitro*. (**a**) Cell viability assay of MES23.5 DA neurons after 6-OHDA (100 mM) treatment of different time points. (**b**) Cell viability assay of MES23.5 DA neurons after treatment with 6-OHDA (100 mM, 6 h) followed by GDNF (100 ng/ml) of different time points. (**c**) Cell counting with trypan blue staining after 6-OHDA (100 mM) treatment of different time points. (**d**) Cell counting with trypan blue staining after 6-OHDA (100 mM, 6 h) followed by GDNF (100 ng/ml) treatment of different time points. (**e**) Apoptosis rate analysis of MES23.5 DA neurons by flow cytometry after treatment with 6-OHDA (100 mM, 6 h) followed by GDNF (100 ng/ml, 12 h). (**f**) Expression of TH protein in DA neurons after treatment with 6-OHDA (100 mM, 6 h) followed by GDNF (100 ng/ml, 12 h). *P<0.05 *versus* control group, **P<0.01 *versus* control group, ^#^*P*<0.05 *versus* the 6-OHDA group. Bar graphs are shown as means±S.E.M. (*n*=3). The statistical analysis was carried out using one-way ANOVA followed by *post hoc* Newman–Keuls and *post hoc* Dunnett's tests

Moreover, we observed protective effects of GDNF on damaged MES23.5 DA cell line *in vitro*. The CCK8 assay results showed that cell viability decreased to approximately 50% after 6 h of 6-OHDA treatment and then increased after 12 h of GDNF rescue (Figures 2a and b). Similarly, cell counting with trypan blue staining and flow cytometry analyses both indicated that the cell apoptosis rate was about 50% at 6 h after 6-OHDA treatment and decreased after 12 h GDNF treatment (Figures 2c and d). The *in vitro* results (6-OHDA 6 h followed by GDNF 12 h) were similar with that for the *in vivo* experiments (6-OHDA 2 w followed by GDNF 1 w). Moreover, we also investigated that TH expression significantly increased after GDNF rescue in damaged MES23.5 DA neurons (Figure 2f). Collectively, these results suggest that GDNF efficiently protects DA neurons damaged by 6-OHDA *in vivo* and *in vitro*.

### GDNF upregulates Six2 expression in 6-OHDA-damaged DA neurons

**Figure 3:**
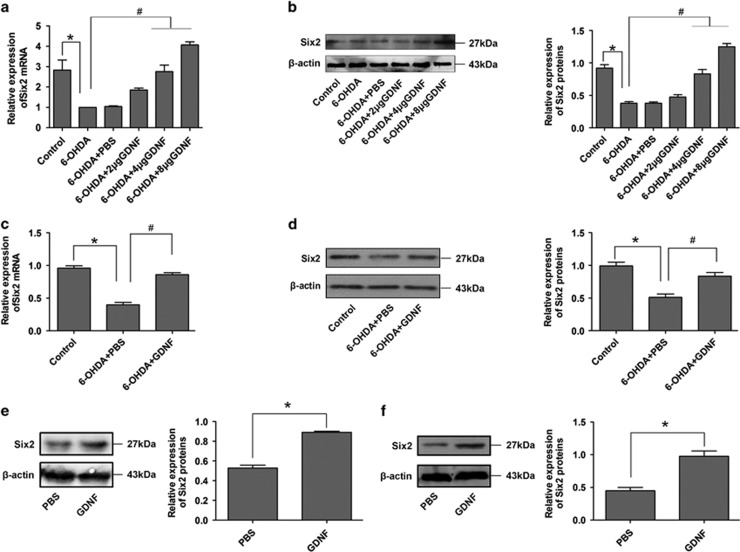
Six2 increased in damaged DA neurons after GDNF rescue *in vivo* and *in vitro*. (**a** and **b**) The mRNA and protein expressions of Six2 in the SN of PD rats *in vivo* models after GDNF rescue (16 *μ*g 6-OHDA for 2 w followed by different doses of GDNF for 1 d). (**c** and **d**) The mRNA and protein expressions of Six2 in damaged MES23.5 DA neuron models after GDNF rescue (100 mM 6-OHDA for 6 h followed by 100 ng/ml GDNF for 12 h). *P<0.05 *versus* control group, ^#^*P*<0.05 *versus* the 6-OHDA group. Bar graphs are shown as means±S.E.M. (*n*=3). The statistical analysis was carried out using one-way ANOVA followed by *post hoc* Newman–Keuls tests. (**e**) The protein expressions of Six2 in the SN of PD rats *in vivo* models after GDNF treatment (8 *μ*g GDNF for 1 d). (**f**) The protein expressions of Six2 in cultured MES23.5 DA neurons after GDNF treatment (100 ng/ml GDNF for 12 h). *P<0.05 *versus* PBS group. Bar graphs are shown as means±S.E.M. (*n*=3). The statistical analysis was carried out using unpaired *t*-tests

In order to detect whether Six2 expression was regulated by GDNF in damaged DA neurons, we assessed Six2 mRNA and protein expressions in the SN of PD rats after GDNF rescue. Increased Six2 levels were highest in the 8 *μ*g GDNF group (Figures 3a and b). We also analysed Six2 expression changes in damaged MES23.5 DA neurons after GDNF rescue. The results revealed that Six2 was obviously increased (Figures 3c and d), which was consistent with the *in vivo* findings. We further detected whether GDNF could increase the Six2 protein level even without 6-OHDA challenge, the results indicated that the Six2 protein levels also increased after GDNF treatment both *in vivo* and *in vitro* (Figures 3e and f). Taken together, these results suggest that Six2 are involved in the GDNF-mediated protection of damaged DA neurons.

### Six2 knockdown attenuates the protective effects of GDNF in damaged DA neurons

**Figure 4:**
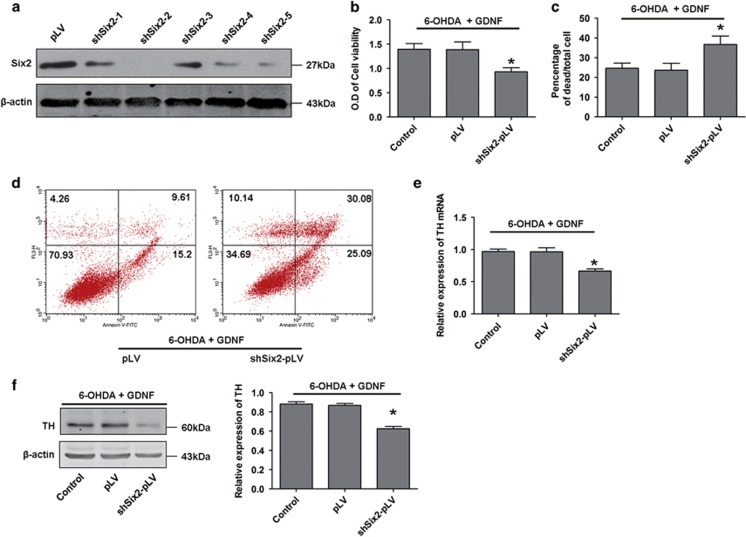
Knockdown of Six2 decreased the protective effects of GDNF on damaged DA neurons. (**a**) Identification of candidate shRNA-Six2 in MES23.5 DA neurons. (**b**–**d**) Cell viability analysis, cell counting after trypan blue staining and flow cytometry apoptosis rate analysis in Six2-knockdown MES23.5 DA neurons after treatment with 6-OHDA (100 mM, 6 h) followed by GDNF (100 ng/ml, 12 h). (**e** and **f**) mRNA and protein expressions of TH in Six2-knockdown MES23.5 DA neurons after treatment with 6-OHDA (100 mM, 6 h) followed by GDNF (100 ng/ml, 12 h). *P<0.05 *versus* control group. Bar graphs are shown as means±S.E.M. (*n*=3). The statistical analysis was carried out using one-way ANOVA followed by *post hoc* Newman–Keuls tests

To test whether Six2 mediates the protection of GDNF in injured DA neurons, a lentivirus-mediated knockdown strategy was utilised to diminish Six2 expression. An shRNA (shSix2-2-pLV) targeting Six2 decreased Six2 expression in MES23.5 DA neurons by approximately 90% (Figure 4a). We further found that the viability of cells infected with shSix2-pLV was reduced compared with pLV-infected MES23.5 DA neurons after GDNF rescue (Figure 4b). Cell counting with trypan blue staining showed that the dead rate of cells infected with shSix2-pLV was higher than that of control cells (Figure 4c). Similarly, flow cytometry analysis also showed that the apoptosis rate of cells infected with shSix2-pLV was higher than that of control cells (Figure 4d). In addition, the mRNA and protein expression levels of TH were also reduced in the shSix2-pLV group (Figures 4e and f). These results suggest that the transcription factor Six2 mediates the protective effects of GDNF on damaged DA neurons.

### Six2 overexpression enhances the protective effects of GDNF in damaged DA neurons

**Figure 5:**
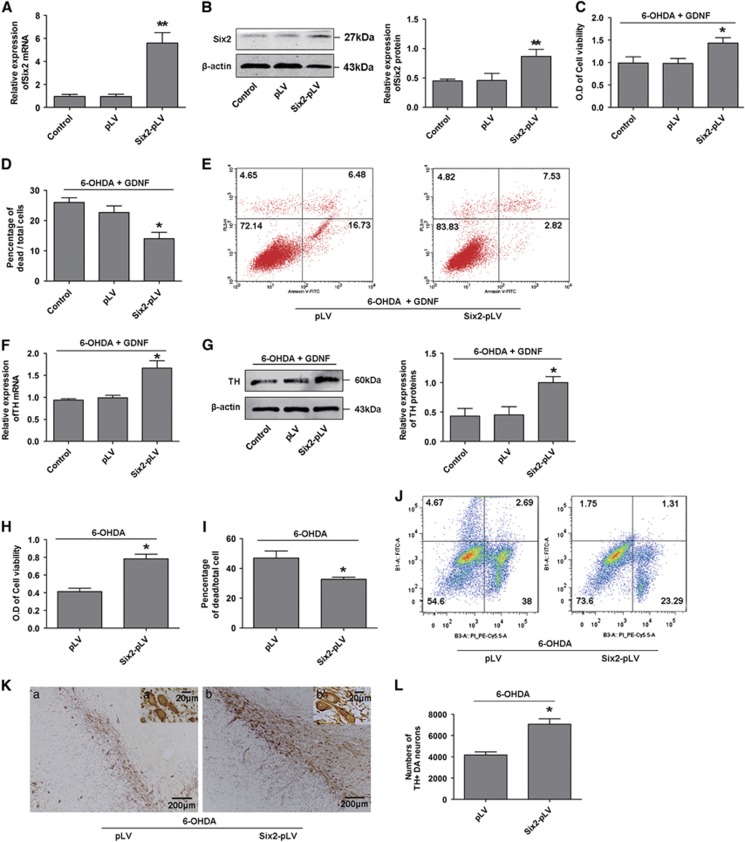
Overexpression of Six2 increased the protective effects of GDNF on damaged DA neurons. (**A** and **B**) The mRNA and protein expressions of Six2 in Six2-overexpressed MES23.5 DA neuron models. (**C**–**E**) Cell viability, cell counting after trypan blue staining and flow cytometry apoptosis rate analysis of Six2-overexpressed MES23.5 DA neuron models after treatment with 6-OHDA (100 mM, 6 h) followed by GDNF (100 ng/ml, 12 h). (**F**) Expression of TH mRNA in Six2-overexpressed MES23.5 DA neuron models after treatment with 6-OHDA (100 mM, 6 h) followed by GDNF (100 ng/ml, 12 h). (**G**) Expression and quantification of TH protein in Six2-overexpressed MES23.5 DA neuron models after treatment with 6-OHDA (100 mM, 6 h) followed by GDNF (100 ng/ml, 12 h). *P<0.05 *versus* control group, **P<0.01 *versus* control group. Bar graphs are shown as means±S.E.M. (*n*=3). The statistical analysis was carried out using one-way ANOVA followed by *post hoc* Dunnett's tests. (**H**) Cell viability analysis of Six2-overexpressed MES23.5 DA neuron models after treatment with 6-OHDA (100 mM, 6 h). (I) Cell counting after trypan blue staining analysis of Six2-overexpressed MES23.5 DA neuron models after treatment with 6-OHDA (100 mM, 6 h). (**J**) Flow cytometry apoptosis rate analysis of Six2-overexpressed MES23.5 DA neuron models after treatment with 6-OHDA (100 mM, 6 h). (**K**) Immunohistochemistry analysis of TH^+^ DA neuron after Six2 overexpression in SN of PD rats *in vivo* models (16 *μ*g 6-OHDA for 2 w). (a) pLV group, (b) Six2-pLV group; scale bars (in a and b) equals 200 *μ*m. a' and b' are higher magnification images in a-b, scale bars equals 20 *μ*m. (L). Counting of the above TH^+^ DA neurons in the SN of PD rats *in vivo* models after Six2 overexpression. * *P<0.05 versus* pLV group. Bar graphs are shown as means±S.E.M. (*n*=3). The statistical analysis was carried out using unpaired *t*-tests

To further demonstrate that Six2 mediates the protective effects of GDNF, we generated stably Six2 overexpression MES23.5 cells. Real-time PCR and western blot analysis demonstrated that Six2 was obviously increased in the Six2 overexpression group (Figures 5A and B). Importantly, damaged MES23.5 cell viability increased after GDNF rescue in the Six2 overexpression group compared with the control group (Figure 5C). Cell counting with trypan blue staining indicated that the dead rate of cells infected Six2-pLV was lower than that of control cells (Figure 5D). In accordance with the above findings, flow cytometry analyses indicated that the apoptosis rate of cells infected Six2-pLV decreased than that of control cells (Figure 5E). In addition, TH expression was significantly increased (Figures 5F and G). We further detected whether Six2 overexpression alone could reduce 6-OHDA toxicity in the absence of GDNF, results showed that MES23.5 cell viability increased in the Six2 overexpression group compared with the control group (Figure 5H), the dead rate of cells decreased in Six2 overexpression group (Figure 5I), and the apoptosis rate of cells also decreased in Six2 overexpression group (Figure 5J). In addition, the *in vivo* experiments indicated that the numbers of TH^+^ neurons were significantly increased in the Six2 overexpression group in SN of PD rats (Figures 5K and L). Thus, our findings strongly indicate that Six2 is involved in the protective effects of GDNF on DA neurons.

### Six2 mediates GDNF protection by upregulating Smurf1 expression

**Figure 6:**
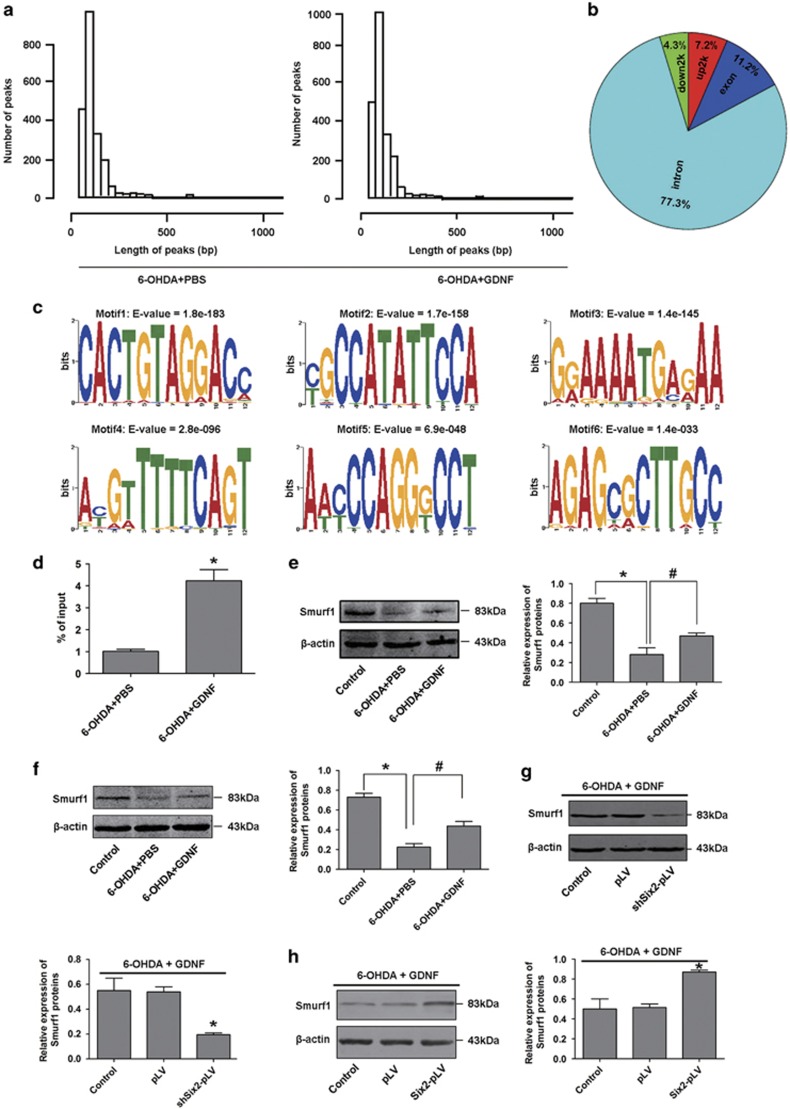
Six2 mediated the protection of GDNF on DA neurons by upregulating Smurf1 expression. (**a**) Peaks of Six2 occupancy in genomic fragments in MES23.5 DA neuron models after treatment with 6-OHDA (100 mM, 6 h) followed by GDNF (100 ng/ml, 12 h). (**b**) Loci distribution of Six2-bound differential expressed target genes in MES23.5 DA neuron models after treatment with 6-OHDA (100 mM, 6 h) followed by GDNF (100 ng/ml, 12 h). The word 'down2k' means 2000 bp downstream from the transcription termination site of the genes, 'up2k' means 2000 bp upstream from the transcription start site of the genes, 'exon' means exon region of the genes, 'intron' means intron region of the genes. (**c**) Potential candidate motifs prediction of Six2 in MES23.5 DA neuron models after treatment with 6-OHDA (100 mM, 6 h) followed by GDNF (100 ng/ml, 12 h). (**d**) Chromatin immunoprecipitation assay with anti-Six2 antibody for Smurf1 in MES23.5 DA neuron models after treatment with 6-OHDA (100 mM, 6 h) followed by GDNF (100 ng/ml, 12 h), ChIP DNA was quantified by qPCR and normalised to input. (**e**) The protein expression and quantitative analysis of Smurf1 in MES23.5 DA neuron models after treatment with 6-OHDA (100 mM, 6 h) followed by GDNF (100 ng/ml, 12 h). (**f**) The protein expression and quantitative analysis of Smurf1 in the SN of PD rats *in vivo* models after GDNF rescue (16 *μ*g 6-OHDA for 2 w followed by 100 ng/ml GDNF for 1 d). (**g**) Expression and quantification of Smurf1 protein in Six2-knockdown MES23.5 DA neuron models after treatment with 6-OHDA (100 mM, 6 h) followed by GDNF (100 ng/ml, 12 h). (**h**) Expression and quantification of Smurf1 protein in Six2-overexpressed MES23.5 DA neuron models after treatment with 6-OHDA (100 mM, 6 h) followed by GDNF (100 ng/ml, 12 h). *P<0.05 *versus* the control group, ^#^*P*<0.05 *versus* the 6-OHDA group. Bar graphs are shown as means±S.E.M. (*n*=3). The statistical analysis was carried out using *t*-test and one-way ANOVA followed by *post hoc* Newman–Keuls tests and *post hoc* Dunnett's tests

**Figure 7:**
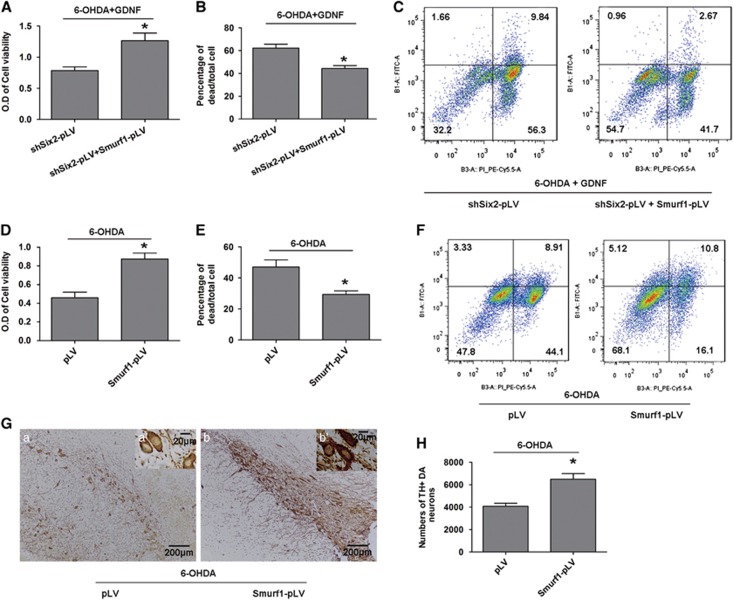
Smurf1 overexpression could rescue the injured DA neurons. (**A**) Cell viability analysis of Smurf1-overexpressed MES23.5 DA neuron models with downregulated Six2 after treatment with 6-OHDA (100 mM, 6 h) followed by GDNF (100 ng/ml, 12 h). (**B**) Cell counting after trypan blue staining of Smurf1-overexpressed MES23.5 DA neuron models with downregulated Six2 after treatment with 6-OHDA (100 mM, 6 h) followed by GDNF (100 ng/ml, 12 h). (**C**) Flow cytometry apoptosis rate analysis of Smurf1-overexpressed MES23.5 DA neuron models with downregulated Six2 after treatment with 6-OHDA (100 mM, 6 h) followed by GDNF (100 ng/ml, 12 h). *P<0.05 versus shSix2-pLV group. Bar graphs are shown as means±S.E.M. (*n*=3). The statistical analysis was carried out using unpaired *t*- tests. (**D**) Cell viability analysis of Smurf1-overexpressed MES23.5 DA neuron models after treatment with 6-OHDA (100 mM, 6 h). (**E**) Cell counting after trypan blue staining analysis of Smurf1-overexpressed MES23.5 DA neuron models after treatment with 6-OHDA (100 mM, 6 h). (**F**) Flow cytometry apoptosis rate analysis of Smurf1-overexpressed MES23.5 DA neuron models after treatment with 6-OHDA (100 mM, 6 h). (**G**) Immunohistochemistry analysis of TH^+^ DA neuron after Smurf1 overexpression in SN of PD rats *in vivo* models (16 *μ*g 6-OHDA for 2 w). (a) pLV group, (b) Smurf1-pLV group; scale bars (in a and b) equals 200 *μ*m. a' and b' are higher magnification images in a and b, scale bars equals 20*μ*m. (**H**) Counting of the above TH^+^ DA neurons in the SN of PD rats *in vivo* models after Smurf1 overexpression. *P<0.05 *versus* pLV group. Bar graphs are shown as means±S.E.M. (*n*=3). The statistical analysis was carried out using unpaired *t*-tests

Our results clearly demonstrate that Six2 is critical for regulating GDNF protection. For an unbiased assessment of the plausible mechanisms of Six2, we performed a genome-wide ChIP-seq analysis for Six2 in MES23.5 DA neurons. The peaks of Six2 occupancy were often broad, with some around 1 kb (Figure 6a). We successfully detected 384 differential genes between DA neurons treated with 6-OHDA followed by GDNF group and DA neurons treated with 6-OHDA group (Supplementary Table 1). Of the Six2-binding sites, 7.2% were located in the region of 2000 bp upstream from transcription start site of the 384 differential genes (Figure 6b). We also performed *de novo* motif discovery to identify specific sequences bound by Six2 (Figure 6c), among these differentially expressed genes, Six2 directly bound to the promoter CAGCTG sequence of smad ubiquitylation regulatory factor 1 (Smurf1). Smurf1 was significantly upregulated after GDNF rescue by ChIP-qPCR analysis (Figure 6d), the protein expression of Smurf1 also increased after GDNF rescue both *in vivo* and *in vitro* by western blot analysis (Figures 6e and f). We further found that Smurf1 expression decreased in cells infected with shSix2-pLV and increased in Six2 overexpression cells after GDNF rescue (Figures 6g and h). We also found that Smurf1 overexpression in 6-OHDA- and GDNF-treated MES23.5 cells with downregulated Six2 could increase cell viability by CCK8 analysis (Figure 7A), decrease the dead rate of cells by cell counting with trypan blue staining (Figure 7B) and decrease the apoptosis rate of cells by flow cytometry analyses (Figure 7C). In addition, we further detected whether Smurf1 overexpression alone could reduce 6-OHDA toxicity in the absence of GDNF, results showed that MES23.5 cell viability increased in the Smurf1 overexpression group (Figure 7D), the dead rate of cells decreased in Smurf1 overexpression group (Figure 7E) and the apoptosis rate of cells also decreased in Smurf1 overexpression group (Figure 7F). In addition, the *in vivo* experiments indicated that the numbers of TH^+^ neurons were significantly increased in the Smurf1 overexpression group in SN of PD rats (Figures 7G and H). These results suggest that Six2 mediates the protective effects of GDNF on damaged DA neurons by upregulating Smurf1.

### Smurf1 mediates GDNF protection by degrading p53

**Figure 8:**
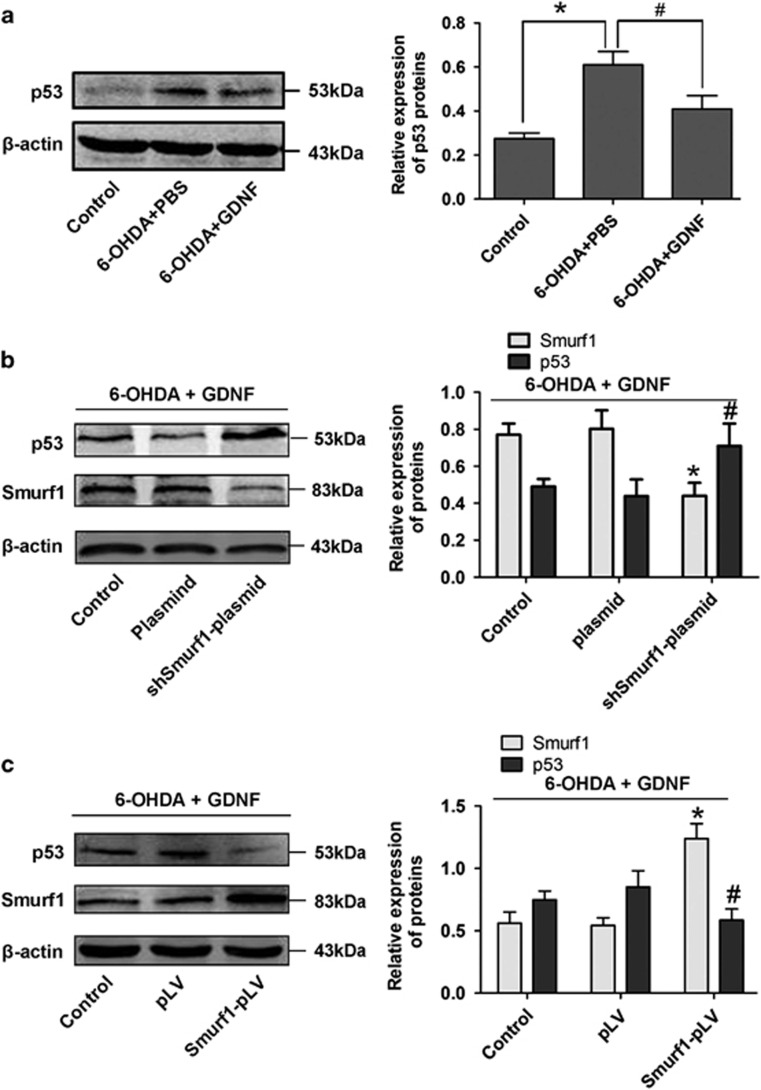
Smurf1 mediated the protection of GDNF on DA neurons by degrading p53. (**a**) The protein expression and quantitative analysis of p53 in the SN of PD rats *in vivo* models after GDNF rescue (16 *μ*g 6-OHDA for 2 w followed by 8 *μ*g GDNF for 1 d). (**b**) The protein expression and quantitative analysis of Smurf1 and p53 in Smurf1-knockdown MES23.5 DA neuron models after treatment with 6-OHDA (100 mM, 6 h) followed by GDNF (100 ng/ml, 12 h). (**c**) Expression and quantification of Smurf1 and p53 protein in Smurf1-overexpressed MES23.5 DA neuron models after treatment with 6-OHDA (100 mM, 6 h) followed by GDNF (100 ng/ml, 12 h). The data were from at least three independent experiments in each case. *P<0.05 *versus* control group of Smurf1, ^#^*P*<0.05 *versus* control group of p53. Bar graphs are shown as means±S.E.M. The statistical analysis was carried out using one-way ANOVA followed by *post hoc* Dunnett's tests

Previous study showed that Smurf1 could promote the degradation of p53.^18^ We first detected the protein expression of p53 after GDNF rescue, the results showed that the p53 protein levels decreased after GDNF rescue in SN of PD rats (Figure 8a). To further test whether Smurf1 mediates the protection of GDNF by degrading p53 in injured DA neurons, a Smurf1 shRNA plasmid (shSmurf1-plasmid) decreased Smurf1 expression in MES23.5 cells by approximately 50%. We further found that the protein expression levels of p53 were increased in the shSmurf1-plasmid group after GDNF rescue (Figure 8b). In addition, we also generated stably Smurf1 overexpression MES23.5 cells. Western blot analysis demonstrated that p53 obviously reduced in the Smurf1 overexpression (Smurf1-pLV) group after GDNF rescue (Figure 8c). These results suggest that the Smurf1 mediates the protective effects of GDNF on damaged DA neurons by degrading the p53 expression.

### Six2 knockdown and overexpression attenuates and increases the protective effects of GDNF in SN of PD rats, respectively

**Figure 9:**
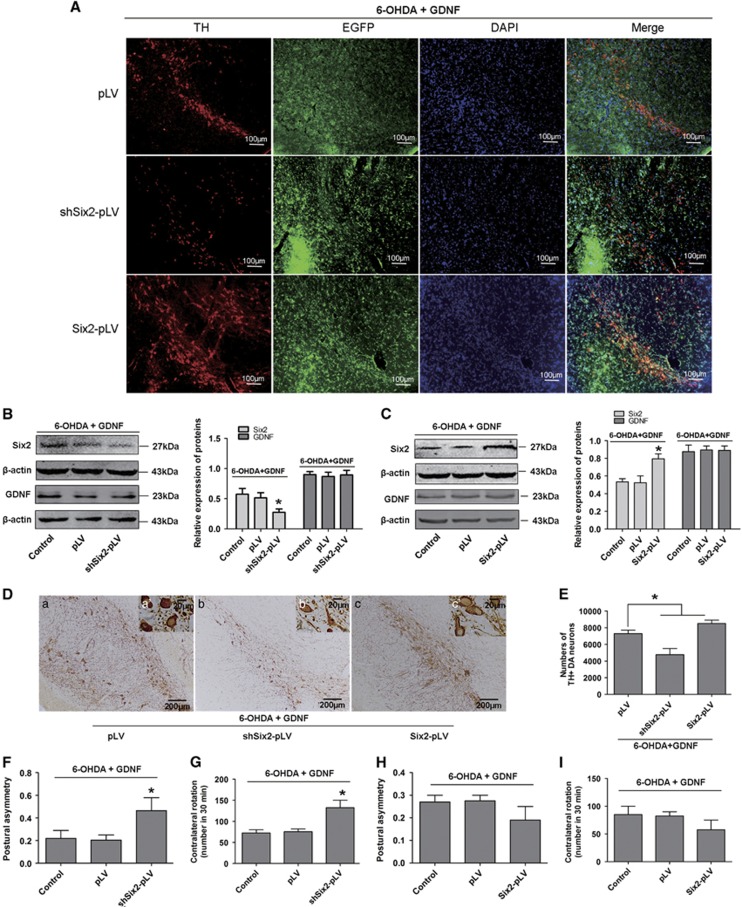
Six2 knockdown and overexpression attenuates and increases the protective effects of GDNF in SN of PD rats, respectively. (**A**) Immunofluorescence staining analysis showed the co-localisation of EGFP and TH in DA neurons in SN of PD rats after GDNF rescue (16 *μ*g 6-OHDA for 2 w followed by 8 *μ*g GDNF for 1 w) (red: TH; green: pLV, Six2-pLV and shSix2-pLV, respectively; blue: DAPI). Scale bars: 100 *μ*m. (**B**) Expression and quantification of Six2 and GDNF in SN of PD rats infected with shSix2-pLV after GDNF rescue (16 *μ*g 6-OHDA for 2 w followed by 8 *μ*g GDNF for 1 d). (**C**) Expression and quantification of Six2 and GDNF in SN of PD rats infected with Six2-pLV after GDNF rescue (16 *μ*g 6-OHDA for 2 w followed by 8 *μ*g GDNF for 1 d). (**D**) Immunohistochemistry analysis of TH^+^ DA neuron after GDNF rescue in Six2 overexpression group in SN of PD rats (16 *μ*g 6-OHDA for 2 w followed by 8 *μ*g GDNF for 1 w). (a) pLV group, (b) shSix2-pLV group, (c) Six2-pLV group; scale bars (in a–c) equals 200 *μ*m. a'–c' are higher magnification images in a and b, scale bars equals 20 *μ*m. (**E**) Counting of the above TH^+^ DA neurons in the SN of PD rats after GDNF rescue. (**F** and **G**) Postural asymmetry tests and apomorphine-induced rotation tests in PD rats infected with shSix2-pLV after GDNF rescue (16 *μ*g 6-OHDA for 2 w followed by 8 *μ*g GDNF for 1 w). (**H** and **I**) Postural asymmetry tests and apomorphine-induced rotation tests in PD rats infected with Six2-pLV after GDNF rescue (16 *μ*g 6-OHDA for 2 w followed by 8 *μ*g GDNF for 1 w). *P<0.05 *versus* control or pLV group. Bar graphs are shown as means±S.E.M. (*n*=8). The statistical analysis was carried out using one-way ANOVA followed by *post hoc* Dunnett's tests

To further demonstrate whether Six2 mediates the protective effects of GDNF on PD rats, we injected shSix2-pLV or Six2-pLV encoded EGFP into SN of PD rats, and then investigated the motor ability after GDNF rescue. Immunofluorescence staining analysis showed these viral-encoded EGFP was, respectively, expressed in TH^+^ neurons in SN of PD rats after GDNF rescue (Figure 9A). Western blot analyses demonstrated that Six2 was obviously decreased in the Six2 knockdown group and increased in the Six2 overexpression group, while GDNF expression were consistent among different groups in SN of PD rats after GDNF rescue (Figures 9B and C). The numbers of TH^+^ neurons were significantly increased in the Six2 overexpression group, and decreased in the Six2 knockdown group in SN of PD rats after GDNF rescue (Figures 9D and E). The posture asymmetry experiment indicated that the frequency of head turning into the lesioned side decreased after Six2 knockdown, and apomorphine-induced rotation increased after Six2 knockdown (Figures 9F and G). Although overexpression of Six2 could promote a little motor ability, but there was no statistical significance (Figures 9H and I). These results indicated that Six2 is involved in the protective effects of GDNF on PD rat models.

## Discussion

GDNF has been shown to ameliorate neurodegeneration in neurotoxin-induced rats and nonhuman primate models of PD;^19, 20, 21^ however, the underlying molecular mechanisms remain largely unknown. Our results are the first evidence that Six2 is at least partially responsible for the protective effects of GDNF in damaged DA neurons. Furthermore, we showed that Smurf1 was directly regulated by Six2 after GDNF rescue, and this inhibited the apoptosis of damaged DA neurons.

GDNF has strong protective effects on DA neurons in the SN of PD models. Previous reports showed that injection of single high doses of GDNF 1 or 3 months after the toxic insult induced upregulation of TH expression and promoted the motor ability of PD models obviously.^22, 23^ Our findings provided additional evidence that single doses of GDNF 2 weeks after 6-OHDA lesion rescued the damaged DA neurons and improved the motor ability of PD rats. This hypothesis is reinforced by the fact that GDNF has been shown to reverse toxin-induced injury to DA neurons.^24, 25^ In this study, we also showed the TH mRNA and protein expression increased 1 day after GDNF injection. A previous study showed that injection of ^125^I-labelled GDNF into striatum, the obvious fluorescence in SN could be detected 6 h after injection, and lasted for several days.^26^ Long-time surveillance showed that the numbers of TH^+^ DA neurons increased from 2 to 21 days in cultured rat embryo midbrain cells treated with GDNF than in control groups.^2^ In addition, the motor activity of rats increased from 2 to 10 days after GDNF injection, the apomorphine-induced rotation behaviour increased most obviously 1 week after GDNF injection.^25, 27^ According to the above studies, we detected the TH mRNA and protein expression after 1 day of GDNF injection, and detected the numbers of TH^+^ DA neurons and behavioural tests after 1 week of GDNF injection in this study. These findings provided additional evidence that GDNF has strong protective effects on damaged DA neurons.

The transcription factor Six2 has been suggested to be involved in kidney development,^13, 28^ and Six2 downregulation results in renal mesenchymal stem cell apoptosis.^16^ Our *in vivo* and *in vitro* analysis showed that Six2 mRNA and protein expression was upregulated in damaged DA neurons after GDNF rescue. These results imply that Six2 mediates GDNF protection. Indeed, knockdown and overexpression experiments *in vitro* confirmed that Six2 mediates the protective anti-apoptotic effect of GDNF, and our *in vivo* analysis also demonstrated that Six2 could protect damaged DA neurons and improve the motor ability of PD rats. Other studies showed that Six1, another member of the SIX family, played critical roles in protecting neurons from apoptosis.^29, 30, 31^ Six1 may also have potential protective functions in DA neurons similar to Six2 because of its similar conserved sequence. Now, the related experiments about Six1 are under way in our laboratory and we wish it would be finished as quickly as possible.

Cell apoptosis occurs via the activation or inhibition of apoptosis-associated factors. Several factors including caspases, p53, and the ubiquitin proteasome system modulate cell apoptosis in damaged DA neurons.^5, 32, 33, 34^ A previous study showed that overexpression of dominant-negative mutants of caspase-3, -7, and -9 could block the death of embryonic DA neurons induced by deprivation of GDNF.^35^ Other studies showed that GDNF/RET signalling were required for normal mitochondrial function and morphology, and blocking caspase-6 could inhibit the death of GDNF-deprived DA neurons.^36, 37^ In addition, overexpression of Nurr1 could reverse the blockade of GDNF signalling, which was induced by deprivation of *α*-synuclein in SN DA neurons.^38^ In this study, although we have shown that Six2 could mediate the protective anti-apoptotic effect of GDNF, the factors that are directly modulated by Six2 after GDNF rescue have not been identified. In this study, ChIP-seq enabled us to investigate the underlying molecular mechanisms governing Six2-mediated protection by GDNF in damaged DA neurons at the genome level. Our results indicated that Smurf1 was targeted by Six2. Smurf1 belongs to the Nedd4 (neuronal precursor cell-expressed developmentally downregulated 4) family of HECT-type E3 ligases and has critical roles in regulating cell growth, migration, and autophagy.^39^ A previous study showed that Smurf1 could promote the degradation of p53,^18^ which is one of the key effectors promoting apoptosis.^40, 41^ In this study, our results also indicated that p53 was degraded by Smurf1 in damaged DA neurons after GDNF treatment. Our results provided additional evidence that the apoptosis of damaged DA neurons could be rescued by GDNF. Taken together, these findings demonstrate that the Six2 is a critical factor that mediates GDNF protection of damaged DA neurons by directly activating Smurf1, which could further degrade p53.

In summary, our results demonstrate that Six2 is a critical regulator of GDNF's ability to inhibit apoptosis of damaged DA neurons by upregulating the expression of Smurf1, which could further degrade p53. These findings provide new important evidence for the protective mechanism of GDNF, which could be useful in identifying potential drug targets for injured DA neurons.

## Materials and Methods

### Animals and cell culture

Adult male (230–250 g) Sprague Dawley rats were provided by Xuzhou Medical College. They were housed under controlled conditions (temperature 23±2 °C and illumination 12:12 h light–dark cycle) with standard diet and water *ad libitum*. Animal housing and treatment of the rats were performed in accordance with the Guidelines of the Ethical Committee for Use of Laboratory Animals.

MES23.5 DA neurons and 293T cells were obtained from the Shanghai Institutes for Cell Resource Center at the Chinese Academy of Sciences (Shanghai, China). All cell lines were cultured in Dulbecco's modified Eagle's medium, supplemented with 10% foetal bovine serum (Hyclone, Logan, UT, USA), 100 units/ml penicillin, and 0.1 mg/ml streptomycin (Invitrogen, Carlsbad, CA, USA) in a 5% CO_2_ atmosphere at 37 °C.

### Reagents and antibodies

The 6-OHDA (H116) and GDNF (G1401) were purchased from Sigma (St. Louis, MO, USA). Rabbit anti-Six2 (SAB2103006), rabbit anti-GDNF antibody (SAB1401150), mouse anti-tyrosine hydroxylase (TH) (T2928), and rabbit anti-Smurf1 antibody (S8449) were also from Sigma. The rabbit anti-Six2 antibody (sc-135274) was from Santa Cruz (St. Louis, MO, USA).The rabbit anti-p53 antibody (BS3736) was from Bioworld Technology (Shanghai, China). The plasmid mini kit (74104) and midi kit (12143) were from Qiagen (Venlo, The Netherlands).

### Constructing of PD rat models using 6-OHDA

The Sprague Dawley rats were anaesthetised with intraperitoneal sodium pentobarbital (50 mg/kg) and placed in the stereotaxic apparatus (bite bar at −3.3 mm). Damage was induced by unilateral stereotaxic injections of 6-OHDA (16 *μ*g total, 4 *μ*g/*μ*l with 2 *μ*l per site) into the left striatum at two coordinates: (i) 0.7 mm anterior and 3 mm lateral to the bregma and 7 mm ventral to the cranial surface and (ii) 0.7 mm anterior and 3 mm lateral to bregma and 5 mm ventral to the cranial surface. The sham-operated rats received injections containing vehicle (0.1% ascorbate in 0.9% saline) at the same coordinates. All injections were performed using a 26-gauge Hamilton syringe connected to an infusion minipump (Hamilton Co., Reno, NV, USA), at a rate of 1 *μ*l/min. After the injection, the syringe was left in place for 5 min and slowly retracted. Two weeks after 6-OHDA injection, the PD rats were screened using behavioural tests. Then, the GDNF was injected into the left striatum of the PD rat models.

### Injection of GDNF into left striatum of PD rat models

This experiment was divided into six groups: (i) Control group: the normal rats (*n*=20); (ii) 6-OHDA group: the rats received left striatum injection of 6-OHDA (*n*=20); (iii) 6-OHDA+ phosphate buffered saline (PBS) group: the PD rats received left striatum injection of PBS (*n*=20); (iv) 6-OHDA+2 *μ*g GDNF: the PD rats received left striatum injection of 2 *μ*g GDNF (*n*=20); (v) 6-OHDA+4 *μ*g GDNF: the PD rats received left striatum injection of 4 *μ*g GDNF (*n*=20); (vi) 6-OHDA+8 *μ*g GDNF: the PD rats received left striatum injection of 8 *μ*g GDNF (*n*=20). One day after GDNF injection, 5 rats in every group were killed and the left sides of SN were processed for real-time polymerase chain reaction (qPCR) analysis, and 5 rats in every group were killed and the left sides of SN were processed for western blot analyses. One week after GDNF injection, the other 10 rats in every group were subjected to behavioural tests, and then, these rats were anaesthetised with sodium pentobarbital and transcardially perfused for immunohistochemistry analyses.

### Behavioural analysis

The following behavioural tests were carried out 2 weeks after 6-OHDA injection for screening the PD rats, and 1 week after GDNF injection for detecting the effect of GDNF on PD rats.

*Postural asymmetry:* Postural asymmetry was analysed using the elevated body swing test as described previously.^42, 43^ The rats were examined for lateral movements with their bodies suspended by their tail 10 cm above the testing table. The frequency of head turning contralateral to the lesioned side (ipsilateral side) was evaluated in 20 continuous trials. Computing method was: (contralateral turns – ipsilateral turns)/total turns × 100%.

*Apomorphine-induced rotation analysis:* Contralateral rotations of each rat were recorded after subcutaneously injection of apomorphine (0.5 mg/kg in normal saline containing 0.01% ascorbic acid) to confirm the dopamine depletion in nigrostriatal system. The rotation behaviour was counted over a period of 60 min. The results were expressed in rotations/30 min.

### Immunohistochemistry

As described previously,^43^ after behavioural tests, 10 rats in every group were anaesthetised and perfused through the left ventricle via an ascending aortic cannulation. According to Paxinos & Watson's The Rat Brain in Stereotaxic Coordinates, we used a stereotaxic apparatus to identify the brain segments containing the SN, which were consecutively sectioned on a microtome in the coronal plane at 6 *μ*m thickness. After blocking for nonspecific binding with 10% donkey serum albumin, these sections were incubated, respectively, with anti-TH antibody and biotinylated horse anti-mouse IgG, followed by the Elite avidin-biotin complex. The reaction was completed with 3'-diaminobenzidine, nickel II sulphate, and H_2_O_2_. Then, these sections were mounted on gelatin-coated slides, dehydrated through graded alcohol, cleared in xylene, and cover-slipped with cytoseal for cell counting.

### Stereology

The numbers of TH^+^ DA neurons in the SNpc (−4.7 to −6.3 mm from bregma) on the injured side were counted under a light microscope.^44^ The first sampling item was taken at random from the frontal part of the SNpc, every sixth section (6 *μ*m thickness) was selected. Sampling was carried out using the Olympus C.A.S.T.-Grid system (Olympus Albertslund, Denmark A/S, Denmark). The SNpc was carefully outlined to exclude other subdivisions of the substantia nigra and the ventral tegmental area by using a × 4 objective. The total number of TH^+^ neurons was analysed using a × 100 Planapo oil immersion objective with a 1.4 numerical aperture. Counting frame (60 um × 60 um) was superimposed on the image of tissue sections. TH^+^ neurons were counted only when present completely or partially inside the frame and when they did not touch any of the red exclusion lines, neurons which touched green inclusion lines were counted. Neurons counted in all sections of the SNpc were summed to give a total number of TH^+^ neurons.

### MES23.5 DA neurons treated with 6-OHDA and GDNF

The MES23.5 cell lines treated with 6-OHDA (100 mM) for different times (30 min, 1 h, 3 h, 6 h, 12 h, and 24 h) were selected for cell counting kit-8 assay (CCK8), trypan blue staining cell viability assay, real-time PCR, and western blot analysis. The MES23.5 DA neurons treated with 6-OHDA (100 mM) for 6 h followed by GDNF (100 ng/ml) for different times (0 h, 3 h, 6 h, 12 h, and 24 h) were also selected for CCK8 assays, cell counting with trypan blue staining, real-time PCR, and western blot analysis.

### Real-time PCR analysis

Total RNA from the tissue samples and cell lines was extracted using high pure RNA isolation kits (Roche Applied Science, Indianapolis, IN, USA). RNA concentration and quality were determined with a Nanodrop ND-1000 spectrophotometer (NanoDrop Technologies, Wilmington, DE, USA) and gel analysis. Then, the RNA was reversely transcribed to cDNA using transcriptor first strand cDNA synthesis kit (Roche Applied Science). The expression of Six2 and TH genes was assayed using the SYBR green PCR master mix (Roche Applied Science). The mRNA data were normalised to *β*-actin. The primers for Six2, TH, and *β*-actin were as follows:

Six2-forward, 5′- GAGGAGACCAGCTACTGCTTCAA-3′,

Six2-reverse, 5′- TTGTGGCTACTGGAATTGGAGTTCT-3′

TH-forward, 5′- TCTATGCTACCCATGCCTGCC-3′,

TH-reverse, 5′- AAATCACGGGCGGACAGTAGAC-3′

*β*-actin-forward, 5′- CACCCGCGAGTACAACCTTC-3′,

*β*-actin-reverse, 5′- CCCATACCCACCATCACACC-3′.

### Western blot analysis

As described previously,^45^ total protein from the tissue samples and cell lines was extracted. Equal amounts of protein samples were separated by sodium dodecyl sulphate/polyacrylamide gel electrophoresis and then transferred onto nitrocellulose membranes (Amersham Pharmacia Biotech, Little Chalfont, UK). After blocking with 3% (w/v) bovine serum albumin for 3 h, the membranes were incubated with primary antibodies and then probed with goat anti-rabbit IR-Dye 670 or 800 cw-labelled secondary antibody. Membranes were imaged using a LiCor Odyssey scanner (Pleasanton, CA, USA). The western blot data were normalised to *β*-actin.

### Establishment of the MES23.5 DA neurons stably expressing shRNA-Six2

Five different targeted sequences homologous to Six2 were designed using the lentiviral expression vector (pLV-H1-EF1a-Bsd). Target sites were as follows: shSix2-1 sense strand, 5′-CCAAGGAAAGGGAGAACAA-3′ shSix2-2 sense strand, 5′-GCTACTGCTTCAAGGAAAA-3′ shSix2-3 sense strand, 5′-AGAATGAAAGCGTGCTCA-3′ shSix2-4 sense strand, 5′-CAGCCAACCTCGTGGACCT-3′ and shSix2-5 sense strand, 5′-GCAACTTCCGCGAGCTCTA-3′. These plasmid DNAs transcribed shRNA with loop sequences of 5′-CTTCCTGTCAGA-3′. The negative control sequence was shScramble sense strand, 5′-GACTTCATAGGCGCATGC-3′. The constructed vector plasmid was transfected into 293T cells together with the packaging plasmids using Lipofectamine 2000 (Invitrogen); the supernatants were collected after 48 h for virus purification. The purified virus containing shSix2-pLV was transfected into MES23.5 cells. After 96 h, cells were passaged and cultured using medium containing puromycin at a final concentration of 2 *μ*g/*μ*l. After seven passages when there was almost no cell death, the MES23.5 cell line stably expressing shRNA-Six2 was established. The cells were harvested for western blot to confirm Six2 knockdown. The cell lines with high knockdown efficiency were treated with 6-OHDA (100 mM) for 6 h followed by GDNF (100 ng/ml) for 12 h, and then selected for CCK8 assays, cell counting with trypan blue staining, flow cytometry analysis, real-time PCR, and western blot analysis.

### Establishment of the MES23.5 DA neurons stably expressing Six2 (or Smurf1)

MES23.5 cells were harvested to extract total RNA. Reverse transcription and amplification of Six2 (or Smurf1) were carried out using Six2 (or Smurf1) upstream and downstream primers, and we then constructed a lentiviral expression vector (pLV-H1-EF1a-Bsd). After sequencing, purified plasmids without mutations and deletions were transfected into 293T cells together with packaging plasmids, as described above. The general protocols included transfecting MES23.5 cells using purified virus and selecting MES23.5 stably expressing Six2 (or Smurf1) using medium containing puromycin. The stably transfected MES23.5 cells were treated with 6-OHDA (100 mM) for 6 h followed by GDNF (100 ng/ml) for 12 h and analysed. The primers of Six2 and Smurf1 were as follows:

Six2: forward, 5′-GCGGAATTCATGTCCATGCTGCCCACCTTC-3′

reverse, 5′-GCGGTCGACGGAGCCCAGGTCCACGAGGTT-3′.

Smurf1: forward, 5′-GCGGAATTCATGTCGAACCCCGGGACCCG-3′

reverse, 5′-GCGGTCGACTCACTCCACGGCAAAGCCACA-3′.

### Smurf1 shRNA plasmids transfection

Smurf1 shRNA plasmid (sc-41674-SH) was from Santa Cruz Biotechnology (Santa Cruz de la Sierra, Bolivia), this plasmid was transfected into MES23.5 cells using lipofectamine 2000 (Invitrogen). Forty-eight hours after transfection, the transfected cells were treated with 6-OHDA (100 mM) for 6 h followed by GDNF (100 ng/ml) for 12 h, and the samples were harvested for western blot analysis.

### Cell viability assay

Cell viability assay was performed using a cell counting kit-8 assay (CCK8; Dojindo Laboratories, Shanghai, China) and a trypan blue staining cell viability assay kit (Beyotime, Jiangsu, China).

### Evaluation of cell apoptosis by flow cytometry analysis

Cells were harvested using 0.25% trypsin and then washed with 0.1 M PBS. After centrifugation at 800 × *g* for 5 min, cells were treated with 100 *μ*l binding buffer and then stained with 2 *μ*l propidium iodide and 2 *μ*l annexin V-fluorescein isothiocyanate for 15 min. In this study, an annexin-V-FLUOS staining kit (Roche Applied Science) was used to assess cell apoptosis, we used the BD FACSCalibur flow cytometry (Becton, Dickinson and Company, Franklin Lakes, NJ, USA) and the MACSQuant flow cytometry (Miltenyi Biotechnology, Bergisch Gladbach, Germany) to examine the cell apoptosis.

### ChIP-seq and ChIP-qPCR

MES23.5 DA neurons were treated with 6-OHDA (100 mM) for 6 h followed by GDNF (100 ng/ml) for 12 h; 12 h after GDNF treatment, cells were fixed in 1% formaldehyde for 10 min, followed by quenching with glycine and rinsing with cold PBS. Afterwards, cells were collected and lysed. The nuclei were released and sonicated into 200–700-base pair (bp) DNA fragments. Aliquots of chromatin were incubated overnight with 5 mg anti-Six2 antibody or control rabbit immunoglobulin G (rIgG, sc-2027) (Santa Cruz Biotechnology, Santa Cruz, CA, USA). The chromatin-antibody complexes were pulled down with prewashed Dyna beads (Invitrogen), washed, and eluted. DNA fragments associated with Six2 or control antibodies were eluted and purified. Input genomic DNA was obtained through similar elution and purification procedures. Prepared DNA was processed for ChIP-seq or ChIP-qPCR. The data from ChIP-seq were analysed by Motif analysis. Real-time qPCRs were performed with the primer pairs as follows:

Smurf1: forward, 5′-CTCACTGGCATAGGGTTCCT-3′

reverse, 5′-TACAGGTGTGCTGGTGAGTT-3′.

### Substantia nigra lentivirus infection to measure Six2 expression and the motor ability of PD rats after GDNF treatment

The adult adult male (230–250 g) Sprague Dawley rats were randomly divided into three groups: (i) pLV groups (*n*=11), in which the rats received a left substantia nigra injection of pLV-EGFP and left striatum injection of 6-OHDA followed by 8 *μ*g GDNF; (ii) Six2-pLV groups (*n*=11), in which the rats received a left substantia nigra injection of Six2-pLV-EGFP and left striatum injection of 6-OHDA followed by 8 *μ*g GDNF; (iii) shSix2-pLV groups (*n*=11), in which the rats received a left substantia nigra injection of shSix2-pLV-EGFP and left striatum injection of 6-OHDA followed by 8 *μ*g GDNF.

The rats were anesthetized with chloralic hydras (10%) and placed in a stereotaxic frame. Burr holes were drilled to permit unilateral stereotaxic injection of 4 *μ*l Six2-pLV or shSix2-pLV or pLV (the final title was 6 × 10^7^ infectious units/ml) at a rate of 1 *μ*l/min into the left SN (stereotaxic coordinated from bregma: anteroposterior:−5.3 mm from bregma, mediolateral:−2.3 mm, and dorso-ventral:−8.3 mm below the surface of the dura). The 6-OHDA lesions were induced as above (see Materials and Methods: Constructing of PD rat models using 6-OHDA), which was injected immediately following substantia nigra pLV delivery. Two weeks after 6-OHDA injection, the apomorphine-induced rotation analysis was used to screen the PD rat models.

Then, GDNF was injected into these PD rats (see Materials and Methods: 2.4 GDNF treatment), 1 day after GDNF treatment, the SN of three rats in every group were quickly removed into liquid nitrogen and used for western blot analysis of Six2 expression. One week after GDNF treatment, the other eight rats in each group were subjected to behavioural tests. After behavioural tests, these rats were deeply anesthetized and perfused, sections were made and collected as described above. These sections were incubated with anti-TH antibody and then with the secondary antibodies. Images were captured with a confocal laser scanning microscope (TCS SP2; Leica, Wetzlar, Germany). For display, images were merged and processed with Leica confocal software (TCS SP2 version 2.5). Only minor adjustments of brightness and contrast were made to the entire images.

### Statistical analysis

All data were processed with SPSS 13.0 (SPSS Inc., Chicago, IL, USA). Bar graphs are shown as means±S.E.M. Difference was carried out using *t* test to compare two independent samples, and one-way analyses of variance (ANOVAs) followed by *post hoc* Newman–Keuls tests to compare all pairs of groups or *post hoc* Dunnett's tests for comparing all experimental groups with the control group. *P*<0.05 was considered statistically significant for all tests.

## Abbreviations

GDNF: Glial cell line-derived neurotrophic factor
DA neurons: dopaminergic neurons
PD: Parkinson's disease
SN: substantia nigra
6-OHDA: 6-hydroxydopamine
SIX: sine oculis homeobox
Smurf1: smad ubiquitylation regulatory factor 1
CCK8 assay: cell counting kit-8 assay
QPCR: real-time polymerase chain reaction
ChIP: chromatin immunoprecipitation

## References

[bib1] Kordower JH, Bjorklund A. Trophic factor gene therapy for Parkinson's disease. Move Disord 2013; 28: 96–109. 10.1002/mds.25344 23390096

[bib2] Lin LF, Doherty DH, Lile JD, Bektesh S, Collins F. GDNF: a glial cell line-derived neurotrophic factor for midbrain dopaminergic neurons. Science 1993; 260: 1130–1132. 8493557 10.1126/science.8493557

[bib3] Peng YS, Lai PL, Peng S, Wu HC, Yu S, Tseng TY et al. Glial cell line-derived neurotrophic factor gene delivery via a polyethylene imine grafted chitosan carrier. Int J Nanomedicine 2014; 9: 3163–3174. 25061293 10.2147/IJN.S60465 PMC4085318

[bib4] Perier C, Bove J, Vila M. Mitochondria and programmed cell death in Parkinson's disease: apoptosis and beyond. Antioxid Redox Signal 2012; 16: 883–895. 21619488 10.1089/ars.2011.4074

[bib5] Venderova K, Park DS. Programmed cell death in Parkinson's disease. Cold Spring Harb Perspect Med 2012; 2: a009365. 22908196 10.1101/cshperspect.a009365 PMC3405826

[bib6] Clarkson ED, Zawada WM, Freed CR. GDNF improves survival and reduces apoptosis in human embryonic dopaminergic neurons *in vitro*. Cell Tissue Res 1997; 289: 207–210. 9211823 10.1007/s004410050867

[bib7] Zawada WM, Zastrow DJ, Clarkson ED, Adams FS, Bell KP, Freed CR. Growth factors improve immediate survival of embryonic dopamine neurons after transplantation into rats. Brain Res 1998; 786: 96–103. 9554968 10.1016/s0006-8993(97)01408-x

[bib8] Helt CE, Hoernig GR, Albeck DS, Gerhardt GA, Ickes B, Reyland ME et al. Neuroprotection of grafted neurons with a GDNF/caspase inhibitor cocktail. Exp Neurol 2001; 170: 258–269. 11476591 10.1006/exnr.2001.7709

[bib9] Li X, Peng C, Li L, Ming M, Yang D, Le W. Glial cell-derived neurotrophic factor protects against proteasome inhibition-induced dopamine neuron degeneration by suppression of endoplasmic reticulum stress and caspase-3 activation. J Gerontol A Biol Sci Med Sci 2007; 62: 943–950. 17895431 10.1093/gerona/62.9.943

[bib10] Cao JP, Wang HJ, Yu JK, Liu HM, Gao DS. The involvement of NF-kappaB p65/p52 in the effects of GDNF on DA neurons in early PD rats. Brain Res Bull 2008; 76: 505–511. 18534259 10.1016/j.brainresbull.2008.03.007

[bib11] Garcez RC, Le Douarin NM, Creuzet SE. Combinatorial activity of Six1-2-4 genes in cephalic neural crest cells controls craniofacial and brain development. Cell Mol Life Sci 2014; 71: 2149–2164. 24061537 10.1007/s00018-013-1477-z PMC11113736

[bib12] Self M, Lagutin OV, Bowling B, Hendrix J, Cai Y, Dressler GR et al. Six2 is required for suppression of nephrogenesis and progenitor renewal in the developing kidney. EMBO J 2006; 25: 5214–5228. 17036046 10.1038/sj.emboj.7601381 PMC1630416

[bib13] Senanayake U, Koller K, Pichler M, Leuschner I, Strohmaier H, Hadler U et al. The pluripotent renal stem cell regulator SIX2 is activated in renal neoplasms and influences cellular proliferation and migration. Hum Pathol 2013; 44: 336–345. 22995329 10.1016/j.humpath.2012.05.021

[bib14] Wang CA, Drasin D, Pham C, Jedlicka P, Zaberezhnyy V, Guney M et al. Homeoprotein Six2 promotes breast cancer metastasis via transcriptional and epigenetic control of E-cadherin expression. Cancer Res 2014; 74: 7357–7370. 25348955 10.1158/0008-5472.CAN-14-0666 PMC4268359

[bib15] Yamamoto-Shiraishi Y, Kuroiwa A. Wnt and BMP signaling cooperate with Hox in the control of Six2 expression in limb tendon precursor. Deve Biol 2013; 377: 363–374. 10.1016/j.ydbio.2013.02.023 23499659

[bib16] Zhou P, Chen T, Fang Y, Wang H, Li M, Ma P et al. Down-regulated Six2 by knockdown of neurofibromin results in apoptosis of metanephric mesenchyme cells *in vitro*. Mol Cell Biochem 2014; 390: 205–213. 24573885 10.1007/s11010-014-1971-0

[bib17] Lv X, Mao Z, Lyu Z, Zhang P, Zhan A, Wang J et al. miR181c promotes apoptosis and suppresses proliferation of metanephric mesenchyme cells by targeting Six2 *in vitro*. Cell Biochem Funct 2014; 32: 571–579. 25187057 10.1002/cbf.3052

[bib18] Nie J, Xie P, Liu L, Xing G, Chang Z, Yin Y et al. Smad ubiquitylation regulatory factor 1/2 (Smurf1/2) promotes p53 degradation by stabilizing the E3 ligase MDM2. J Bio Chem 2010; 285: 22818–22830. 20484049 10.1074/jbc.M110.126920 PMC2906273

[bib19] Bilang-Bleuel A, Revah F, Colin P, Locquet I, Robert JJ, Mallet J et al. Intrastriatal injection of an adenoviral vector expressing glial-cell-line-derived neurotrophic factor prevents dopaminergic neuron degeneration and behavioral impairment in a rat model of Parkinson disease. Proc Natl Acad Sci USA 1997; 94: 8818–8823. 9238061 10.1073/pnas.94.16.8818 PMC23145

[bib20] Eberling JL, Kells AP, Pivirotto P, Beyer J, Bringas J, Federoff HJ et al. Functional effects of AAV2-GDNF on the dopaminergic nigrostriatal pathway in parkinsonian rhesus monkeys. Hum Gene Ther 2009; 20: 511–518. 19254173 10.1089/hum.2008.201 PMC2725183

[bib21] Espejo EF, Gonzalez-Albo MC, Moraes JP, El Banoua F, Flores JA, Caraballo I. Functional regeneration in a rat Parkinson's model after intrastriatal grafts of glial cell line-derived neurotrophic factor and transforming growth factor beta1-expressing extra-adrenal chromaffin cells of the Zuckerkandl's organ. J Neurosci 2001; 1: 9888–9895. 10.1523/JNEUROSCI.21-24-09888.2001 PMC6763029 11739596

[bib22] Bowenkamp KE, Hoffman AF, Gerhardt GA, Henry MA, Biddle PT, Hoffer BJ et al. Glial cell line-derived neurotrophic factor supports survival of injured midbrain dopaminergic neurons. J comp neurol 1995; 355: 479–489. 7636027 10.1002/cne.903550402

[bib23] Gash DM, Zhang Z, Ovadia A, Cass WA, Yi A, Simmerman L et al. Functional recovery in parkinsonian monkeys treated with GDNF. Nature 1996; 380: 252–255. 8637574 10.1038/380252a0

[bib24] Hoffer BJ, Hoffman A, Bowenkamp K, Huettl P, Hudson J, Martin D et al. Glial cell line-derived neurotrophic factor reverses toxin-induced injury to midbrain dopaminergic neurons *in vivo*. Neurosci Lett 1994; 182: 107–111. 7891873 10.1016/0304-3940(94)90218-6

[bib25] Hudson J, Granholm AC, Gerhardt GA, Henry MA, Hoffman A, Biddle P et al. Glial cell line-derived neurotrophic factor augments midbrain dopaminergic circuits *in vivo*. Brain Res Bull 1995; 36: 425–432. 7712205 10.1016/0361-9230(94)00224-o

[bib26] Tomac A, Widenfalk J, Lin LF, Kohno T, Ebendal T, Hoffer BJ et al. Retrograde axonal transport of glial cell line-derived neurotrophic factor in the adult nigrostriatal system suggests a trophic role in the adult. Proc Natl Acad Sci USA 1995; 92: 8274–8278. 7667281 10.1073/pnas.92.18.8274 PMC41139

[bib27] Kobayashi S, Ogren SO, Hoffer BJ, Olson L. Dopamine D1 and D2 receptor-mediated acute and long-lasting behavioral effects of glial cell line-derived neurotrophic factor administered into the striatum. Exp Neurol 1998; 154: 302–314. 9878169 10.1006/exnr.1998.6952

[bib28] Brodbeck S, Besenbeck B, Englert C. The transcription factor Six2 activates expression of the Gdnf gene as well as its own promoter. Mech Dev 2004; 121: 1211–1222. 15327782 10.1016/j.mod.2004.05.019

[bib29] Konishi Y, Ikeda K, Iwakura Y, Kawakami K. Six1 and Six4 promote survival of sensory neurons during early trigeminal gangliogenesis. Brain Res 2006; 1116: 93–102. 16938278 10.1016/j.brainres.2006.07.103

[bib30] Plant KE, Anderson E, Simecek N, Brown R, Forster S, Spinks J et al. The neuroprotective action of the mood stabilizing drugs lithium chloride and sodium valproate is mediated through the up-regulation of the homeodomain protein Six1. Toxicol Appl Pharmacol 2009; 235: 124–134. 19101580 10.1016/j.taap.2008.10.019

[bib31] Yajima H, Suzuki M, Ochi H, Ikeda K, Sato S, Yamamura K et al. Six1 is a key regulator of the developmental and evolutionary architecture of sensory neurons in craniates. BMC Bio 2014; 12: 40–57. 24885223 10.1186/1741-7007-12-40 PMC4084797

[bib32] Checler F, Alves da Costa C. p53 in neurodegenerative diseases and brain cancers. Pharmacol Ther 2014; 142: 99–113. 24287312 10.1016/j.pharmthera.2013.11.009

[bib33] Radi E, Formichi P, Battisti C, Federico A. Apoptosis and oxidative stress in neurodegenerative diseases. J Alzheimers Dis 2014; 42: S125–S152. 25056458 10.3233/JAD-132738

[bib34] Rhodes SL, Fitzmaurice AG, Cockburn M, Bronstein JM, Sinsheimer JS, Ritz B. Pesticides that inhibit the ubiquitin-proteasome system: effect measure modification by genetic variation in SKP1 in Parkinsons disease. Environ Res 2013; 126: 1–8. 23988235 10.1016/j.envres.2013.08.001 PMC3832349

[bib35] Yu LY, Saarma M, Arumae U. Death receptors and caspases but not mitochondria are activated in the GDNF- or BDNF-deprived dopaminergic neurons. J Neurosci 2008; 28: 7467–7475. 18650325 10.1523/JNEUROSCI.1877-08.2008 PMC6670859

[bib36] Yu LY, Arumae U. Survival assay of transiently transfected dopaminergic neurons. J Neurosci Methods 2008; 169: 8–15. 18191457 10.1016/j.jneumeth.2007.11.018

[bib37] Meka DP, Müller-Rischart AK, Nidadavolu P, Mohammadi B, Motori E, Ponna SK et al. Parkin cooperates with GDNF/RET signaling to prevent dopaminergic neuron degeneration. J Clin Invest 2015; 125: 1873–1885. 25822020 10.1172/JCI79300 PMC4611569

[bib38] Decressac M, Kadkhodaei B, Mattsson B, Laguna A, Perlmann T, Björklund A. a-Synuclein-induced down-regulation of Nurr1disrupts GDNF signaling in nigral dopamine neurons. Sci Transl Med 2012; 4: 156–163. 10.1126/scitranslmed.3004676 23220632

[bib39] Cao Y, Zhang L. A Smurf1 tale: function and regulation of an ubiquitin ligase in multiple cellular networks. Cell Mol Life Sci 2013; 70: 2305–2317. 23007848 10.1007/s00018-012-1170-7 PMC11113965

[bib40] da Costa CA, Sunyach C, Giaime E, West A, Corti O, Brice A et al. Transcriptional repression of p53 by parkin and impairment by mutations associated with autosomal recessive juvenile Parkinson's disease. Nat Cell Bio 2009; 11: 1370–1375. 19801972 10.1038/ncb1981 PMC2952934

[bib41] Liang ZQ, Li YL, Zhao XL, Han R, Wang XX, Wang Y et al. NF-kappaB contributes to 6-hydroxydopamine-induced apoptosis of nigral dopaminergic neurons through p53. Brain Res 2007; 1145: 190–203. 17368433 10.1016/j.brainres.2007.01.130

[bib42] Borlongan CV, Saporta S, Poulos SG, Othberg A, Sanberg PR. Viability and survival of hNT neurons determine degree of functional recovery in grafted ischemic rats. Neuroreport 1998; 9: 2837–2842. 9760130 10.1097/00001756-199808240-00028

[bib43] Chang CF, Lin SZ, Chiang YH, Morales M, Chou J, Lein P et al. Intravenous administration of bone morphogenetic protein-7 after ischemia improves motor function in stroke rats. Stroke 2003; 34: 558–564. 12574575 10.1161/01.str.0000051507.64423.00

[bib44] Luquin MR, Saldise L, Guillén J, Belzunegui S, San Sebastián W, Izal A et al. Does increased excitatory drive from the subthalamic nucleus contribute to dopaminergic neuronal death in parkinson's disease? Exp Neurol 2006; 201: 407–415. 16806173 10.1016/j.expneurol.2006.04.033

[bib45] Liu HM, Gao J, Miao H, Xiao CH, Sun Y, Du X et al. Influence of aging on the calbindin-D-28k immunoreactive positive dopaminergic neurons in the substantia nigra pars compacta of rats. Neurosci Lett 2010; 468: 3–6. 19857553 10.1016/j.neulet.2009.10.044

